# Turkish validity and reliability of the level of sitting scale in children with cerebral palsy

**DOI:** 10.55730/1300-0144.5621

**Published:** 2023-01-15

**Authors:** Kamile UZUN AKKAYA, Sabiha BEZGİN, Debra FIELD, Bülent ELBASAN

**Affiliations:** 1 Department of Physiotherapy and Rehabilitation, Faculty of Health Sciences, Gazi University, Ankara, Turkey; 2 Department of Physiotherapy and Rehabilitation, Faculty of Health Sciences, Hatay Mustafa Kemal University, Hatay, Turkey; 3 Sunny Hill Health Centre for Children, Vancouver, British Columbia, Canada

**Keywords:** Cerebral palsy, children, reliability, sitting level, validity

## Abstract

**Background/aim:**

The Level of Sitting Scale (LSS) is a valid and reliable classification index that categorizes sitting ability. The aim of this study is to establish the Turkish validity and reliability of the LSS in children with cerebral palsy (CP).

**Materials and methods:**

In total, 165 children (75 girls and 90 boys) between the ages of 4 and 18 years who were diagnosed with CP were included in the study. All children were evaluated by two independent physiotherapists for the interrater reliability analysis of the LSS and were reevaluated 1 week later by the same physiotherapist for the intrarater reliability analysis. The Gross Motor Function Classification System (GMFCS) was used for validity analysis.

**Results:**

The intrarater reliability analyses of the LSS showed an intraclass correlation coefficient of 0.999 (ICC 95% CI [0.999–1]), and the interrater reliability analyses showed an intraclass correlation coefficient of 0.998 (ICC 95% CI [0.998–0.999]). A statistically significant, negative, and strong correlation was found between the GMFCS and the LSS (p < 0.001, r = −0.770).

**Conclusion:**

The Turkish version of the LSS in children with CP is a valid and reliable scale. The Turkish LSS can be used by researchers and clinicians in research and to determine the sitting level of children with CP.

## 1. Introduction

Cerebral palsy (CP) occurs as a result of permanent but nonprogressive damage in the developing brain, in which many physical, mental, emotional, and cognitive problems are seen together [1]. Physical problems continue throughout life as a result of this central nervous system damage and functional limitations vary with growth and development [2–4]. Motor problems associated with CP may include abnormal muscle tone, muscle weakness, postural control deficiencies, primitive reflex patterns, and atypical muscle movements and these may influence a child’s ability to sit, crawl, and walk [5–8].

Sitting is one position that promotes children’s trunk control with purposeful use of their upper extremities as well as engagement in functional activities, and interaction with their environment. Facilitating a stable and well-aligned sitting position has many benefits, such as: maintaining head and trunk control, reaching in different directions, bringing the hands to midline, and being able to grasp objects [9–11]. According to the International Classification of Functioning, Disability and Health (ICF), sitting ability falls within the activity component of the ICF and is valuable to evaluate [12] as one of the many factors affecting function in children with CP [12, 13]. Sitting ability is very important to evaluate especially in children with CP who cannot walk, to assist with treatment planning and decision-making regarding use of a wheelchair and/or other assistive devices [14].

The Level of Sitting Scale (LSS) is a valid and reliable classification index that categorizes sitting ability in children with neuromotor disabilities across eight levels [15]. From the point of view of physiotherapists, it is important to evaluate sitting balance and postural control in children with CP, to determine intervention programs and to choose the equipment best matched to children’s needs [14]. One systematic review of clinical tools used to measure sitting balance in children with CP reported that the LSS is a useful measure [16]. In addition, by classifying sitting ability, the LSS can assist in treatment planning, in deciding the appropriate device and amount of postural support required to increase children’s participation in activities of daily living, in data collection, and to create a common language among health professionals [12, 14, 15, 17]. The LSS has been translated into other language and used in different countries [18, 19]. The validity and reliability study of the Italian version of the LSS in children with CP was conducted by Italian researchers in 2019 [18]. At present, there is no valid and reliable Turkish scale assessing the level of sitting ability. The present study aimed to establish the validity and reliability of the Turkish LSS in children with CP.

## 2. Materials and methods

### 2.1. Participants

We conducted this methodological study between June 2020 and June 2021 in the pediatric rehabilitation unit. Ethical approval for the study was obtained from Gazi University Clinical Research Ethics Committee on 04.02.2020 with ethics committee number 91610558-604.01.02. The families of the children who agreed to participate in the study provided written informed consent. After obtaining permission from the original author of the scale, the LSS was translated into Turkish and culturally adapted in accordance with the internationally recognized method [20].

Children between the ages of 4 and 18 years who were diagnosed with CP by a pediatric neurologist were included in the study. Children who had undergone botulinum toxin injections or any type of surgery in the last 6 months or who did not agree to participate in the study were excluded.

### 2.2. Data collection

Evaluations were made by two physiotherapists with 10 years of experience in pediatric rehabilitation while the children’s parents were present. Just prior to determining the classifications, the families of the children were asked to fill in the demographic information form inquiring about age, height, body weight, and sex of the children. We also recorded whether the children used a wheelchair and/or adaptive sitting devices. To facilitate interrater reliability analysis, the two evaluators independently classified the child’s sitting abilities using the LSS, and they were blinded to each other’s evaluation. Convergent construct validity was estimated by evaluating the relationship between Gross Motor Function Classification System (GMFCS) and LSS values. Rating of GMFCS level was performed by experienced physiotherapists. It took an average of 20 min to evaluate one child.

To facilitate intrarater reliability analysis, LSS evaluations were performed again 1 week later by the same evaluator. The evaluators conducted the LSS assessments after completing training via a free online training module.

#### 2.2.1. Gross Motor Function Classification System (GMFCS)

The GMFCS is a valid and reliable 5-level classification system used to categorize gross motor functional abilities in children with CP. Level 1 indicates the least involvement and level 5 indicates the most severe involvement [21]. Numerous studies have used the GMFCS to describe gross motor abilities of children with CP [22, 23].

#### 2.2.2. Level of Sitting Scale

The LSS is a valid and reliable 8-level classification index designed by a team of researchers and clinicians at Sunny Hill Health Centre for Children [15]. At Level 1, two (or more) adults are required to provide external postural support in order to maintain an upright sitting position for 30 s, while at Levels 2 through 4, the child is able to maintain a sitting position for 30 s with varying degrees of postural support by one adult (respectively at the head, shoulder/chest or pelvis). At level 5, the child is able to sit still for 30 s without the use of their hands or feet for postural support, or adult assistance. For Levels 6 through 8, the child is able to sit independently while moving the trunk at least 20 degrees out of their base of support in one of three directions (respectively forward, sideways, and backward) and reerecting without falling or using their hands for support [14, 15]. The LSS was revised by Field et al. in 2019 and the revised version of the scale was used in our study. The LSS provides a common language for describing sitting ability among researchers, clinicians, clients, and families [14, 15]. It can be used for convenient data collection as well as guiding clinical decision-making purposes regarding therapeutic interventions [14, 15]. It takes approximately 5–10 min to administer and score the LSS. Level 8 indicates higher functioning than Level 1.

#### 2.2.3. Translation procedure

After receiving permission from the original authors, the LSS was translated and culturally adapted according to international guidelines [20]. First, the original scale was translated into Turkish by two independent Turkish-speaking physiotherapists. The Turkish form was evaluated and synthesized upon by the researchers, then was translated back from Turkish to English by two bilingual translators. The text translated into English and the original text were compared by the researchers, and the translated English text was sent to one of the tool developers via e-mail for their opinion. Revisions were made on the Turkish where necessary for cultural adaptations and to increase intelligibility by the other members of the study team, and by this way, language validity was attained [20].

### 2.3. Data analysis

Statistical analyses of the study were performed using the “Statistical Product and Service Solutions” (SPSS) program version 22.0 [24]. Descriptive analyses are presented using mean and standard deviations for normally distributed variables, and median and minimum–maximum values for ordinal variables. Categorical variables are presented as numbers and percentages. Two-way random effect model intraclass correlation coefficients (ICC) with a 95% confidence interval (CI) were used for the intrarater and interrater reliability analysis of the Turkish LSS. Values between 0.41 and 0.60 indicate moderate agreement, values between 0.61 and 0.80 indicate substantial agreement, and values between 0.81 and 1.00 indicate excellent agreement [25]. Additionally, the standard error of measurement (SEM) and minimal detectable change (MDC) were used for reliability analysis. The correlation between the LSS and the GMFCS was estimated using Spearman correlation coefficient. Spearman correlation coefficients were interpreted as the following relationships: ≥0.70, strong; 0.70 to 0.30, moderate; <0.30, weak [26].

## 2. Results

The study included 165 children with CP. The children’s mean age was 8.88 ± 3 years. Their mean height was 120.67 ± 17.62 cm, and their average body weight was 25.96 ± 10.91 kg. The children’s sex, CP subtype, GMFCS and LSS levels, whether they use a wheelchair, whether they use postural supports, and the support type are presented in Table 1.

**Figure 1:**
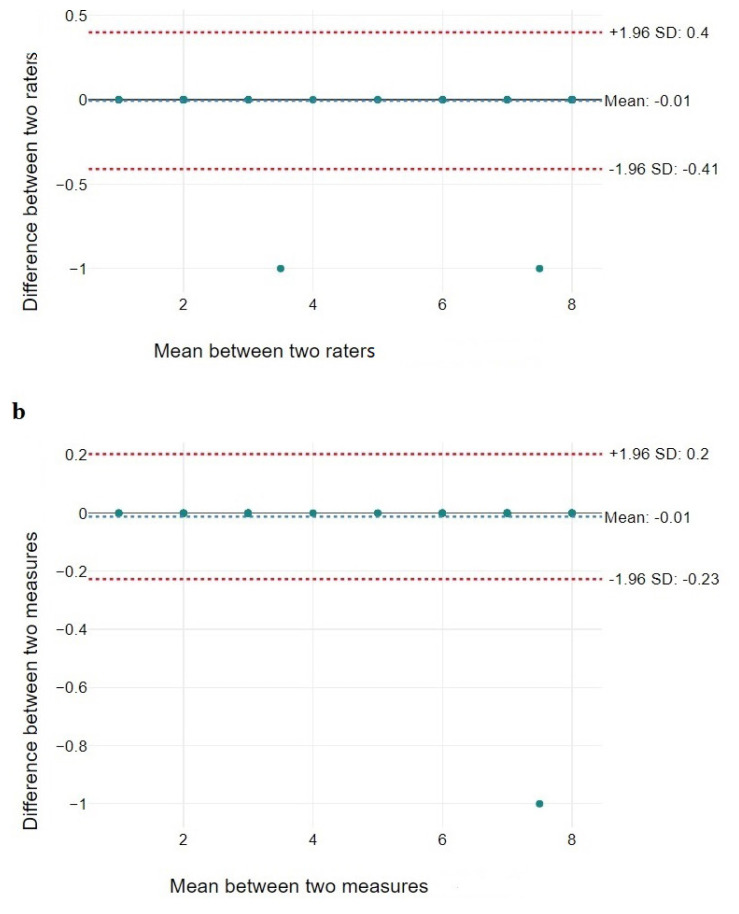
Bland–Altman plots for (a) interrater reliability and (b) intrarater reliability.

Table 2 provides the LSS reliability coefficients (ICC) and the confidence levels (95% CI) among the raters (interrater) (Figure 1a) and by each rater within a 1-week period (intrarater) (Figure 1b). The LSS intrarater reliability ICC was 0.999 and the interrater reliability ICC was 0.998, leading us to conclude that the reliability levels were excellent. SEM and MDC results are presented in Table 2.

A statistically significant, negative, and strong correlation was found between GMFCS and LSS measures (p < 0.001, r = −0.770) (Table 3).

## 3. Discussion

This study aimed to analyze the validity and reliability of the Turkish LSS in children with CP, and we concluded that the Turkish LSS is a valid and reliable measure to classify children’s sitting ability. Sitting position in children with CP can improve trunk control, postural stability and fine motor skills as well as enhancing communication and participation in daily life activities [27, 28]. Although there are Turkish translations of classifications for gross motor function and manual dexterity in children with CP [29, 30], it is noteworthy that this is the first Turkish language scale classifying sitting ability that has evidence supporting its validity and reliability with children who have CP. Both the original English version and the Turkish translation of the LSS categorize a range of sitting abilities. Field et al. [14] reported that the LSS is a valid scale for assessing sitting ability in children with neuromotor impairment. In addition, in this study, it was found that the LSS values of the children and the amount of postural support adaptations required by them were related [14]. In the original study by Fife and colleagues, interrater reliability was reported to have a mean Kappa value of 0.60, mean agreement 69%, while test–retest reliability was mean kappa value 0.55, mean agreement 64% [15]. Although the LSS achieved fair [15] interrater reliability rating and fair [15] test-retest reliability ratings in the initial study, a second study by the developers, which included revisions, reported good interrater reliability rating [17], and good test-retest reliability ratings [17]. We determined that the Turkish version of the LSS had excellent reliability both for interrater (ICC 0.998), and intrarater (ICC 0.999). Our findings are similar to the study by Tofani et al. [18]. Their study found that the Italian LSS had excellent interrater reliability (ICC 0.99), and one-week test-retest reliability (ICC 0.99) in classifying the sitting ability of Italian children with CP. The SEM provides reference data for clinicians to interpret the magnitude of measurement error and decide the actual score for the test or scale used. MDC symbolizes the minimum performance change of an individual rather than bias or measurement error [31, 32]. This study is novel since it provides a comprehensive methodology that SEM and MDC values of LSS were demonstrated for the first time. As a result of these analyses, it was determined that the Turkish version of the LSS is a reliable scale that can be used in children aged 4–18 years with CP.

In our study, the GMFCS was used to determine the convergent construct validity of the LSS. Both GMFCS and LSS are used to classify motor abilities of children with CP, although the GMFCS focuses on the gross motor functions and mobility, whereas the LSS focuses on sitting ability. Our results suggest an inverse relationship where Level I GMFCS represents the highest level of independence, while Level 1 in LSS represents children with the highest degree of dependency in sitting. Mendoza et al. [19] reported that there was a negative correlation between GMFCS and LSS levels in children with CP, and concluded that the GMFCS and the LSS are useful tools for describing the functional abilities and limitations of children with CP, particularly regarding mobility and sitting (respectively). Tofani et al. [18] conducted an Italian study examining the validity and reliability of the LSS and they also examined the relationship between the GMFCS and the LSS to assess construct validity. They also found a very strong negative correlation between the two measures, with lower LSS scores (children who need a high level of adaptive sitting support) being associated with higher values on the GMFCS [18]. Our study showed similarities with these other studies in that we concluded that the Turkish version of the LSS was valid and there was an association between gross motor function and sitting abilities. There is a need for feasible, reliable and valid assessment tools in the rehabilitation of children with CP. Children with CP perform many activities of daily living in a sitting position. It is noteworthy that there is no Turkish version of a scale for sitting. As a result of our study, a common language will be formed for health professionals about the prognosis and development of children with CP at different levels with the evaluations made with Turkish LSS in children with CP.

### 3.1. Limitations

One of the limitations of our study was that the majority of the children participating in the study were children with spastic type CP. In the future, validity and reliability analyses of this classification can be performed in children with diagnoses other than CP in order to broaden its application to a wider population. Another limitation was that different tools were not used to assess trunk and gross motor functions for validity analyses in this study, and only GMFCS was used. The last limitation was the fact that cognitive, visual, and auditory disorders accompanying CP were not assessed and their effects on sitting levels were not examined. Again, this could be evaluated in a future study.

## 4. Conclusion

The Turkish version of the LSS is a valid and reliable scale that can be used in children aged 4–18 years with CP both clinically and for research purposes. The Turkish version of the LSS may be useful to researchers and clinicians to create a common language when determining the sitting level of children with CP. Information from the LSS will assist Turkish-speaking clinicians in planning treatment programs, and determining the amount of external postural support needed to optimize sitting function.

## Figures and Tables

**Table 1:** Demographic information of participants.

	Mean ± SD		Med (min–max)
Age (Years)	8.88 ± 3	9 (4–18)	
Height (cm)	120.67 ± 17.62	121 (83–175)	
Body weight (kg)	25.96 ± 10.91	23 (10–70)	
BMI (kg/m^2^)	17.10 ± 3.04	16.5 (11–25.39)	
		N (165)	%
Sex	Female	75	45.5
	Male	90	54.5
Types of CP	Dyskinetic CP	2	1.2
	Unilateral spastic CP	54	32.7
	Bilateral spastic CP	109	66.1
GMFCS	1	27	16.4
	2	48	29.1
	3	21	12.7
	4	24	14.5
	5	45	27.3
LSS	1	8	4.8
	2	14	8.5
	3	22	13.3
	4	2	1.2
	5	3	1.8
	6	13	7.9
	7	23	13.9
	8	80	48.5
Wheelchair users	No	91	55.2
	Yes	74	44.8
Postural support users	No	95	57.6
	Yes	70	42.4
Types of postural support	No support	95	57.6
	Head/Neck	25	15.2
	Trunk	25	15.2
	Pelvic	15	9.1
	Thigh	5	3
	Total	165	100.0

CP: Cerebral palsy; SD: Standard deviation; Med: Median; Min: Minimum; Max: Maximum; cm: Centimeter; kg: Kilogram; m: Meter; BMI: Body mass index; GMFCS: Gross motor function classification system; LSS: Level of sitting scale.

**Table 2:** Intrarater and interrater reliability of the Level of Sitting Scale.

Intrarater reliability					

	Rater 1	Rater 1	ICC (95% CI)	Paired differences mean (SEM)	MDC
	First assessment	Second assessment			
	Med (min–max)	Med (min–max)			

**LSS**	7 (1–8)	7 (1–8)	**0.999 (0.999–1)**	−0.012 (0.009)	0.025

**Interrater reliability**					
	
	Rater 1	Rater 2	ICC (95% CI)	Paired differences mean (SEM)	MDC
	First assessment	First assessment			
	Med (min–max)	Med (min–max)			

**LSS**	7 (1–8)	7 (1–8)	**0.998 (0.998–0.999)**	−0.006 (0.016)	0.044

LSS: Level of Sitting Scale; Med: Median; Min: Minimum; Max: Maximum;

ICC: Intraclass correlation coefficient; CI: Confidence interval; SEM:

Standard error of mean; MDC: Minimal detectable change (SEM × 1.96 × √2).

**Table 3:** Correlations between LSS and GMFCS.

	Correlations with LSS	
GMFCS	r	**−** **0.770** *
	p	**<0.001**

LSS: Level of Sitting Scale; GMFCS: Gross Motor Function Classification System

* p < 0.001 (Spearman correlation coefficient)

## Abbreviations

CP: Cerebral Palsy
GMFCS: The Gross Motor Function Classification System
ICC: Intraclass Correlation Coefficients
ICF: International Classification of Functioning
LSS: Level of Sitting Scale
SPSS: Statistical Product and Service Solutions
